# Phase I Trial of Triapine–Cisplatin–Paclitaxel Chemotherapy for Advanced Stage or Metastatic Solid Tumor Cancers

**DOI:** 10.3389/fonc.2017.00062

**Published:** 2017-04-04

**Authors:** Charles A. Kunos, Edward Chu, Della Makower, Andreas Kaubisch, Mario Sznol, Susan Percy Ivy

**Affiliations:** ^1^Cancer Therapy Evaluation Program, National Cancer Institute, Bethesda, MD, USA; ^2^University of Pittsburgh Cancer Institute, University of Pittsburgh School of Medicine, Pittsburgh, PA, USA; ^3^Montefiore Medical Center, Bronx, NY, USA; ^4^Yale University School of Medicine, Yale Cancer Center, New Haven, CT, USA

**Keywords:** triapine, cisplatin, paclitaxel, phase I clinical trial, cancer, maximum tolerated dose, cervical cancer, uterine cervix cancer

## Abstract

Ribonucleotide reductase (RNR) is an enzyme involved in the *de novo* synthesis of deoxyribonucleotides, which are critical for DNA replication and DNA repair. Triapine is a small-molecule RNR inhibitor. A phase I trial studied the safety of triapine in combination with cisplatin–paclitaxel in patients with advanced stage or metastatic solid tumor cancers in an effort to capitalize on disrupted DNA damage repair. A total of 13 patients with various previously treated cancers were given a 96-h continuous intravenous (i.v.) infusion of triapine (40–120 mg/m^2^) on day 1, and then 3-h i.v. paclitaxel (80 mg/m^2^) followed by 1-h i.v. cisplatin (50–75 mg/m^2^) on day 3. This combination regimen was repeated every 21 days. The maximum tolerated dose (MTD) for each agent was identified to be triapine (80 mg/m^2^), cisplatin (50 mg/m^2^), and paclitaxel (80 mg/m^2^). Common grade 3 or 4 toxicities included reversible anemia, leukopenia, thrombocytopenia, or electrolyte abnormalities. The combination regimen of triapine–cisplatin–paclitaxel resulted in no objective responses; however, five (83%) of six patients treated at the MTD had stable disease between 1 and 8 months duration. This phase I study showed that the combination regimen of triapine–cisplatin–paclitaxel was safe and provides a rational basis for a follow-up phase II trial to evaluate efficacy and progression-free survival in women with metastatic or recurrent uterine cervix cancer.

## Introduction

Uterine cervix cancers are aggressive gynecological malignancies marked by abdominopelvic lymph node or visceral organ metastases and by poor metastatic disease-specific survival, and up to 80% have been shown to overexpress ribonucleotide reductase (RNR) (1–4). Women with recurrent uterine cervix cancer treated by first-line cisplatin–paclitaxel combination chemotherapy have disease response (46%) more often than other cisplatin–non-platinum combinations (12–31%) (5–9). Adding the humanized vascular endothelial growth factor-neutralizing monoclonal antibody bevacizumab to cisplatin–paclitaxel chemotherapy in the same setting resulted in higher disease response (50%) and a median survival of 18 months (4). However, no curative therapy exists for women with metastatic uterine cervix cancer, and therefore, these women have a significant unmet therapeutic need.

**Figure 1:**
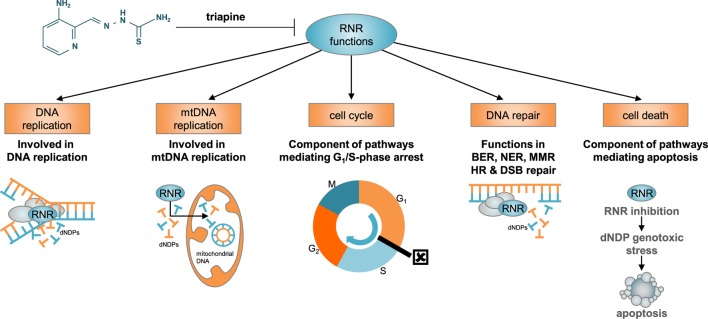
**Besides its classical role in nuclear DNA replication, RNR has diverse functions in other biological processes, including mitochondrial DNA replication, cell cycle regulation, DNA damage repair, and apoptosis**. RNR acts as the primary *de novo* reductase supplying on-demand dNDP DNA precursor pools. In this role, RNR rate limits nuclear and mitochondrial DNA replication. Its payouts are tightly regulated by allosteric feedback (apoptosis effects), by S-phase-dependent M2 subunit expression (cell cycle arrest effects), and by p53 protein–M2b subunit interactions (DNA repair effects). The RNR catalytic mechanism involves a proton-coupled electron transfer, relocating iron-stabilized tyrosyl radicals housed in its small M2 or M2b subunits to radical-based catalytic sites in its large M1 subunits. Pharmacological disruption of the M2 or M2b iron–metal moieties by triapine is sufficient to render RNR inactive. Abbreviations: RNR, ribonucleotide reductase; mtDNA, mitochondrial DNA; BER, base excision repair, NER, nucleotide excision repair; MMR, mismatch repair; HR, homologous recombination; DSB, double-strand brake; dNDP, deoxynucleotide diphosphates.

Based on knowledge of cell cycle controlled expression and activity of RNR, there was, until recently, a fairly simple understanding of the molecular behavior of cancers harboring unchecked RNR like those of the uterine cervix. In dormant (G_0_-phase) or resting (G_1_-phase) cells, when levels of RNR subunit proteins are low, enzyme activity, and therefore nucleotide output, is minimal (10). In replicating cells (S–G_2_-phase) like cancer cells, when RNR subunit proteins are expressed at elevated levels, enzyme activity and nucleotide output are maximal—ultimately regulated by inherent feedback allosteric sites in RNR and not necessarily by levels of enzyme subunit expression (11). The allosteric activity site detects the overall nucleotide concentration and balances *de novo* nucleotide production (12). When RNR activity is high, DNA damage repair is timely, facile, and impacts downstream cell fate decisions that are prosurvival rather than lethal (13–18). Expansion of nucleotide pools during DNA damage has a clear physiological role, and it has been shown that increased nucleotide concentrations as outputs from RNR help cells survive DNA damage (19). Such findings provide a strong rationale for clinical development of new agents that exploit RNR repair and DNA damage responses, especially in cancers with unchecked RNR. Triapine (also known as 3-aminopyridine-2-carboxaldehyde thiosemicarbazone or 3-AP) is a potent inhibitor of RNR with known antiproliferative and cytotoxic effects (13–18). In preclinical studies of uterine cervix cancer cells, triapine potently blocks deoxynucleotide output by RNR after DNA damage, protracts cell cycle arrest at the G_1_–S-phase checkpoint, and leads to unresolved γH2AX foci (i.e., phosphorylated histones flanking DNA double-strand breaks) marking DNA damage (13–15)—all disruptive to normal RNR functions (Figure 1).

Early phase I studies of triapine alone and in combination in patients with metastatic and advanced stage cancers have shown intermittent triapine to have relatively mild toxicity, but continuous intravenous (i.v.) infusion triapine reaches a maximum tolerated dose (MTD) in terms of drug-related toxicities (20–25). Thus, this phase I trial was designed to evaluate whether continuous i.v. infusion triapine could inhibit RNR and potentiate the antitumor effects of cisplatin–paclitaxel with acceptable toxicity levels in patients with metastatic or advanced stage solid tumor cancers refractory to standard therapy or for which no curative therapy existed (http://clinicaltrials.gov number, NCT00016874).

## Materials and Methods

### Study Design and Treatment

VION-015 was an open-label dose-finding trial of i.v. triapine–cisplatin–paclitaxel chemotherapy (Table 1). Patients were enrolled according to a dose escalation schema using a standard Fibonacci 3 + 3 patient cohort phase I trial design. This trial’s design declared a single drug-related adverse event in a three-patient cohort as an event to prompt an additional three patients being accrued (i.e., six patients in the dose level cohort) for confirmation of a 33% rate of attributable toxicity. Accrual continued if no other adverse events were observed (i.e., two or fewer of six patients in the dose level cohort); otherwise, accrual discontinued. The MTD was established when six patients had been treated with less than or equal to one toxic event. Adverse events were scored in this trial according to the Common Terminology Criteria for Adverse Events (version 2.0), which was the criteria used at-the-time this study was conducted. Adverse events indicating dose-limiting toxicity included all severe or life-threatening toxicities, grade 3 non-hematologic toxicities, grade 4 neutropenia lasting more than 3 days or associated with infection, grade 4 thrombocytopenia lasting more than 3 days or associated with clinically significant bleeding, or persistent adverse events of any grade requiring delay of scheduled treatment by more than 2 weeks.

**Table 1:** **Dose escalation and extent of drug exposure**.

Dose level	Triapine dose (mg/m^2^/day)	Paclitaxel dose (mg/m^2)^	Cisplatin dose (mg/m^2)^	No. of new patients	Total no. of patients treated^a^	Total no. course administered
**Triapine day 1 and paclitaxel–cisplatin day 3 q3-week schedule (***n*** = 11)**						
1	40	80	50	4	5	14
2	80	80	50	6	7	26
3	80	80	75	1	1	1
**Triapine day 1 and paclitaxel–cisplatin day 3 q3-week schedule (***n*** = 2)^b^**						
−2	40	80	50	0	0	0
−1	80	80	50	0	0	0
1	120	80	50	2	2	2

*^a^Some patients were treated at more than one dose level*.

*^b^The protocol was amended to introduce a fixed starting dosing of paclitaxel and cisplatin*.

Patients were administered i.v. triapine by 96-h continuous infusion at an initial dose of 40 mg/m^2^ starting on day 1 (Table 1). Triapine infusions were repeated every 21 days. Vion Pharmaceuticals supplied i.v. triapine in 50 mg viscous liquid vials, which were diluted for an ambulatory continuous infusion pump per the manufacturer’s instruction. Administration and i.v. bag sets were exchanged fresh after every 48 h of infusion.

Patients received i.v. paclitaxel over 3 h at a single fixed dose of 80 mg/m^2^ starting on day 3 (Table 1). Commercial paclitaxel was diluted for infusion per the manufacturer’s instruction. Participants received i.v. cisplatin over 1 h between 50 and 75 mg/m^2^ starting on day 3 (Table 1). Commercial cisplatin also was diluted for infusion per the manufacturer’s instruction. In this particular trial, paclitaxel infusion preceded cisplatin infusion. Cisplatin and paclitaxel administrations were also repeated every 21 days.

By definition for this trial, two courses of triapine–cisplatin–paclitaxel constituted one cycle of therapy. Patients were eligible to receive a second or subsequent cycle of therapy if they had stable disease or, a partial or complete response.

### Eligibility and Enrollment

The trial was open to women and men 18 years of age or older with a diagnosis of a measurable metastatic or advanced stage cancer refractory to one or more standard therapies or for which no curative therapy existed. Patients must have had an Eastern Cooperative Oncology Group performance status score of 0 or 1; a life expectancy greater than 3 months; adequate kidney, liver, and bone marrow function as determined by laboratory assessment; and must have been practicing adequate contraception or abstinence. Female patients of childbearing potential must have had a negative pregnancy test within 2 weeks before study drug administration. Patients must not have had active heart disease, moderate-to-severe compromise of pulmonary function, active infections, severe hearing impairment or grade 2 or higher neuropathy, active central nervous system metastases, presence of any other life-threatening illness, prior severe allergic reaction to study agents, or presence of any active bleeding or coagulation disorder. Patients must have had prior treatment for their cancer, had at least 3 weeks of recovery from prior surgery, last dose of chemotherapy, and/or last dose of radiotherapy. Persistent toxicities from prior treatments must not have been greater than grade 1. Patients who had received cisplatin or paclitaxel individually, but not in combination, were eligible. Patients who had received cisplatin–paclitaxel must not have had the pair within the previous 6 months before planned study entry. Patients whose prior therapy included triapine were eligible.

### Assessments

Medical histories, physical examinations including performance status assessment, urinalysis, and laboratory studies, including serum chemistries and complete blood counts were obtained at baseline, before the first dose of each 96-h triapine cycle, once each interval week, and at off-study. Chest radiograph, computed tomography scans of the chest and abdomen (excluding pelvis), and serum tumor markers (i.e., CA-125, CA19-9, CEA) were obtained every two cycles. Magnetic resonance imaging of the brain or bone scan was obtained every two cycles, as clinically indicated for a patient’s disease and status.

### Study Oversight

The principal investigators (Della Makower, Andreas Kaubisch, and Mario Sznol) designed the clinical trial and the dose escalation/dose reduction schema. Vion Pharmaceuticals, the sponsor of the study, supplied triapine and collected all the clinical data, but did not participate in the study design, analysis, or manuscript preparation. An independent quality assurance audit (Prologue Research International) documented in compliance with all applicable regulations during the trial period. All participating sites received institutional review board approval prior to first dose infusion. A manuscript publication team (Charles A. Kunos, Edward Chu, and Susan Percy Ivy) subsequently reviewed, collated, authenticated, and analyzed the trial data for manuscript publication.

### Evaluation of Clinical Activity and Statistical Analysis

Response Evaluation Criteria in Solid Tumors (version 1.0) were applied at baseline and every two treatment cycles for response and for disease progression (26). No statistical analyses were performed.

## Results

### Patients

Patients were eligible for this phase I study from April 2001 through December 2002. A total of 13 patients were enrolled to receive triapine–cisplatin–paclitaxel, and all patients were evaluated with regard to safety. Baseline patient characteristics are listed in Table 1. All patients underwent previous treatment for their advanced stage or metastatic cancer, including surgery (*n* = 6), cytotoxic chemotherapy (*n* = 8), or palliative radiation therapy (*n* = 4). None of the enrolled patients had prior hormonal therapy or immunotherapy. As seen in Table 2, three different dose levels were evaluated.

**Table 2:** **Patient and disease characteristics (*n* = 13)**.

Characteristic	No. of patients	%^a^
**Age (years)**		
30–39	2	15
40–49	1	8
50–59	5	38
60–69	5	38
**Sex**		
Female	8	62
Male	5	38
**Race**		
White	8	62
Black or African-American	3	23
Asian/Pacific Islander	2	15
**Ethnicity**		
Hispanic	4	31
Non-Hispanic	9	69
**Performance status**		
0	5	38
1	8	62
**Disease site**		
Colon	3	23
Uterine cervix	2	15
Bladder	1	8
Bile duct	1	8
Esophageal	1	8
Gastric	1	8
Pyriform sinus	1	8
Small intestine	1	8
Tonsil	1	8
Adenocarcinoma, not specified	1	8

*No., number*.

*^a^May not total 100% due to rounding*.

### Safety

Table 3 lists the most common toxicities observed in all three dose levels of the study. The most frequent adverse events included grade 1 constipation, nausea, or emesis; grade 2 fatigue; grade 3 thrombocytopenia; and reversible grade 3 or 4 anemia (10 [77%] of 13) and leukopenia (8 [62%] of 13). The hematologic toxicity observed in one participant with uterine cervical cancer at the 80 mg/m^2^ dose of triapine, 75 mg/m^2^ dose of cisplatin, and 80 mg/m^2^ dose of paclitaxel prompted enrollment of the next three patients to the next lower dose level. This also led to an amendment of the protocol for an escalated triapine dose preceding a fixed dose of cisplatin and paclitaxel (Table 2). The triapine (80 mg/m^2^)–cisplatin (50 mg/m^2^)–paclitaxel (80 mg/m^2^) dose level was declared the MTD. In six patients treated at the MTD, the attributed grade 3 or 4 toxicity rate was 50% with the main side effects being reversible fatigue, hyperbilirubinemia, anemia, leukopenia, and thrombocytopenia.

**Table 3:** **Adverse events by grade with any relationship to triapine–paclitaxel–cisplatin (*n* = 13)**.^a^

	Grade				
	1	2	3	4	5
	
Toxicity	No.	No.	No.	No.	No.
Allergy/immunology	0	0	0	1	0
Blood/bone marrow (other)	0	3	0	0	0
Anemia	0	1	7	3	0
Febrile neutropenia	0	0	0	0	0
Leukopenia	1	0	2	6	0
Thrombocytopenia	0	1	5	0	0
Cardiovascular	5	0	2	0	0
Constitutional (other)	15	12	4	0	0
Fatigue	5	6	2	1	0
Dermatology/skin	11	3	1	0	0
Endocrine/special senses (other)	4	3	0	0	0
Tinnitus	0	0	0	0	0
Hearing Loss	1	0	0	0	0
Gastrointestinal (other)	16	2	0	0	0
Constipation	6	2	0	0	0
Diarrhea	4	3	0	0	0
Emesis	6	3	3	0	0
Nausea	6	4	2	0	0
Infection	0	0	2	0	0
Metabolic/nutritional	19	6	6	1	0
Creatinine increased	0	0	0	0	0
Hypokalemia	4	0	1	1	0
Hyponatremia	0	0	1	0	0
Musculoskeletal	1	0	0	0	0
Neurology	11	8	1	0	0
Respiratory system (other)	11	1	0	0	0
Dyspnea	1	1	1	0	0
Hypoxia	0	0	1	0	0
Pulmonary embolus	0	0	0	1	0
Renal/genitourinary	2	2	1	0	0
Sexual/reproductive function	0	0	0	0	0
Worst non-hematologic	128	56	28	5	0
Worst hematologic	1	5	14	9	0

*No., number*.

*^a^Patients may have had more than one adverse event*.

Dose reductions of triapine occurred in two patients (15%) after the first course of treatment. There were no dose reductions of cisplatin or paclitaxel. Three patients (23%) discontinued therapy after the first course of treatment. The reasons for treatment discontinuation included skin toxicity in the form of Stevens–Johnson syndrome in one patient, physician preference in one patient, and patient preference in one patient. The median number of treatment cycles was 3 (range, 1–9 courses). No treatment-related deaths occurred while on study. A fatal adverse event occurred in one (8%) patient, with this event being attributed to disease progression within 30 days after receipt of study treatment.

### Efficacy

**Figure 2:**
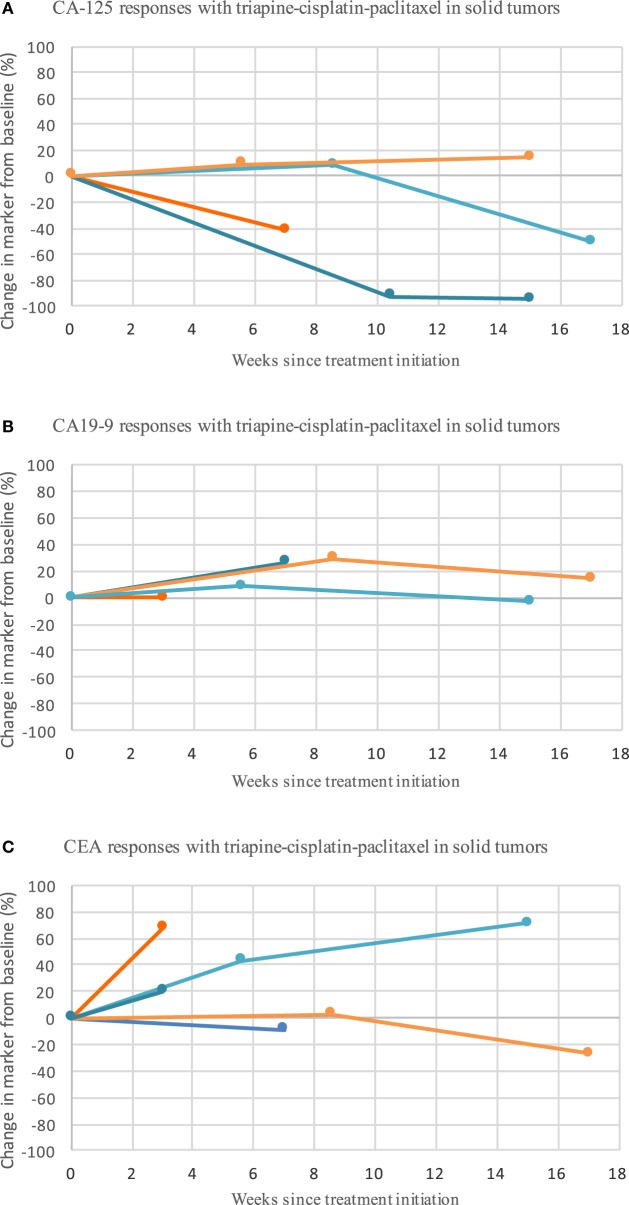
**Triapine–cisplatin–paclitaxel induces mixed biomarker responses in patients with metastatic or advanced stage solid tumor cancers refractory to standard therapy or for which no curative therapy existed**. Shown are the biomarker levels (assessed as the longest linear dimension) over time for CA-125 **(A)**, CA19-9 **(B)**, and CEA **(C)**. Circles mark the posttherapy time point at which biomarker assessment was obtained. CA-125, CA19-9, and CEA did not associate with the pattern or duration of response. Abbreviations: CA-125, cancer antigen-125; CA 19-9, cancer antigen 19-9; CEA, carcinoembryonic antigen.

For patients who received at least two courses of therapy (i.e., one cycle), no objective responses were observed. Stable disease occurred in six (46%) patients. The durations of stable disease response were 1, 2, 2, 5, 7, and 8 months. In the MTD cohort, five patients (of six, 83%) best responses of stable disease were observed. One patient in the MTD cohort had metastatic uterine cervix cancer, and her disease response was determined to be approximately 30% after three therapy cycles (i.e., seven 21-day infusion courses). Her stable disease response lasted 8 months. Progression of disease was recorded in five (38%) patients at initial response assessment. In one (8%) patient with uterine cervix cancer enrolled at the triapine (80 mg/m^2^)–cisplatin (75 mg/m^2^)–paclitaxel (80 mg/m^2^) dose level, symptomatic decline did not permit documentation of disease status, and thus, this patient was listed arbitrarily as having had clinical disease progression. Response was not evaluated after study entry in one (8%) patient. Serum tumor marker responses appear in Figure 2.

## Discussion

This phase I trial found that triapine–cisplatin–paclitaxel was a safe therapeutic option for patients with previously treated advanced stage or metastatic solid tumor cancers. Patients treated using triapine–cisplatin–paclitaxel most often reached a stable disease status, consistent with at-the-time known preclinical (18) and clinical data (20–22) among a broad spectrum of molecularly driven cancer phenotypes, and not just uterine cervix cancers.

In this trial, there was an elevated rate of grade 3 or 4 anemia (77%) after triapine–cisplatin–paclitaxel, possibly as a result of potent iron chelation by triapine (27) without interference in total iron binding capacity (22). This rate of anemia was not observed in contemporary cisplatin–paclitaxel trials [18–25% (5, 28)]. Patients who received triapine–cisplatin–paclitaxel had a similar incidence of grade 3 or 4 leukopenia (62%) in the context of another cisplatin–paclitaxel trial [64% (5)].

Until the results of triapine–cisplatin–radiation trials were reported (23–25), non-randomized early phase trials provided the best evidence base for RNR inhibition by triapine before cytotoxic chemotherapy (29–32). An assumption made in the 90s was that cancer cells gained from overproduced RNR or from overactive RNR from relaxed allosteric regulation (33), and that sustained pharmacological blockade of RNR would kill cancer cells (18). Two trials involved continuous infusion triapine infusion for sustained RNR inhibition, and unfortunately, yielded no partial or complete disease responses by standard criteria (21, 22). Other triapine-agent combination trials scheduling triapine before cytotoxic chemotherapy also resulted in few (8 [9%] of 88 participants) disease responses (29–32). This trial of triapine before cisplatin–paclitaxel also found no partial or complete disease responses by standard measurement criteria or by serum tumor markers (when available). Later preclinical data found that triapine scheduled before cytotoxic agents blocks overall RNR activity for cytostatic effect, stimulates overproduction of RNR for restored activity 18–24 h later, facilitates DNA repair, and ultimately may be prosurvival in effect (32). Another discovery that the highly electrophilic platinum ion (Pt^3+^) rapidly depletes the concentration of free triapine over a long incubation period in cell-free medium (34), thereby attenuating triapine’s RNR-blocking activity, cautions sequencing triapine before cisplatin–paclitaxel. But for the converse sequence, triapine given after DNA-damaging cisplatin or radiation inhibits overall RNR activity for cytotoxic effect, halts nucleotide supply by RNR when most vital to repair of damaged DNA, protracts DNA repair, and therefore, is highly cytotoxic (13–15, 32). As an example, triapine–cisplatin–radiation trials show a 96% disease response rate (23–25). Thus, protracted nucleotide demand (e.g., by damaging DNA) unmet by nucleotide supply (e.g., by inhibiting RNR) becomes intolerable and cell death occurs. Sequencing effects of triapine–paclitaxel on the mitotic spindle and late cell cycle-phase nucleotide demands have not been explored and warrant further preclinical study.

Interpretative limitations for this trial include accrual of few participants, lowering an ability to assess safety and therapeutic response in disease-specific subgroups such as women with metastatic uterine cervix cancer, infrequent biomarker collection to inform response efficacy, potential investigator bias due to relatively arbitrary changes in triapine–cisplatin–paclitaxel trial logistics following observed toxicity, and recall bias due to a very delayed time from trial closure to trial publication.

In summary, this phase I trial provides evidence that the combination of the RNR inhibitor triapine and the cytotoxic chemotherapies cisplatin and paclitaxel may be given together safely in patients with metastatic or advanced stage solid tumor cancers. Future studies should consider the use of oral triapine for RNR blockade optimally integrated into a second-line cisplatin–paclitaxel chemotherapy regimen for women with metastatic uterine cervix cancer.

## Ethics Statement

This study was carried out in accordance with the recommendations of the Montefiore Medical Center and of the Weill Cornell Medical College with written informed consent from all subjects. All subjects gave written informed consent in accordance with the Declaration of Helsinki. The protocol was approved by the Institutional Review Boards of the Montefiore Medical Center and of the Weill Cornell Medical College.

## Author Contributions

All authors contributed to the concept, data collection, and writing of this manuscript.

## Conflict of Interest Statement

The authors declare that the research was conducted in the absence of any commercial or financial relationships that could be construed as a potential conflict of interest. The reviewer, ER, declared a shared affiliation, though no other collaboration, with one of the authors, MS, to the handling editor, who ensured that the process nevertheless met the standards of a fair and objective review.

## References

[1] Kunos CA Radivoyevitch T Kresak A Dawson D Jacobberger J Yang B Elevated ribonucleotide reductase levels associate with suppressed radiochemotherapy response in human cervical cancers. Int J Gynecol Cancer (2012) 22(9):1463–9. 10.1097/IGC.0b013e318270577f 23051959 PMC3481180

[2] Kunos CA Winter K Dicker AP Small W Jr Abdul-Karim FW Dawson D Ribonucleotide reductase expression in cervical cancer: a radiation therapy oncology group translational science analysis. Int J Gynecol Cancer (2013) 23(4):615–21. 10.1097/IGC.0b013e31828b4eb5 23552804 PMC3662019

[3] Monk BJ Sill MW Burger RA Gray HJ Buekers TE Roman LD. Phase II trial of bevacizumab in the treatment of persistent or recurrent squamous cell carcinoma of the cervix: a gynecologic oncology group study. J Clin Oncol (2009) 27(7):1069–74. 10.1200/JCO.2009.21.8909 19139430 PMC2667811

[4] Tewari KS Sill MW Long HJ III Penson RT Huang H Ramondetta LM Improved survival with bevacizumab in advanced cervical cancer. N Engl J Med (2014) 370(8):734–43. 10.1056/NEJMoa1309748 24552320 PMC4010094

[5] Rose PG Blessing JA Gershenson DM Mcgehee R. Paclitaxel and cisplatin as first-line therapy in recurrent or advanced squamous cell carcinoma of the cervix: a gynecologic oncology group study. J Clin Oncol (1999) 17(9):2676–80. 10.1200/JCO.1999.17.9.2676 10561341

[6] Morris M Blessing JA Monk BJ Mcgehee R Moore DH. Phase II study of cisplatin and vinorelbine in squamous cell carcinoma of the cervix: a gynecologic oncology group study. J Clin Oncol (2004) 22(16):3340–4. 10.1200/JCO.2004.12.006 15310778

[7] Muggia FM Blessing JA Mcgehee R Monk BJ. Cisplatin and irinotecan in squamous cell carcinoma of the cervix: a phase II study of the Gynecologic Oncology Group. Gynecol Oncol (2004) 94(2):483–7. 10.1016/j.ygyno.2004.05.018 15297192

[8] Farley J Sill MW Birrer M Walker J Schilder RJ Thigpen JT Phase II study of cisplatin plus cetuximab in advanced, recurrent, and previously treated cancers of the cervix and evaluation of epidermal growth factor receptor immunohistochemical expression: a Gynecologic Oncology Group study. Gynecol Oncol (2011) 121(2):303–8. 10.1016/j.ygyno.2011.01.030 21329967 PMC3081894

[9] Miller DS Blessing JA Ramondetta LM Pham HQ Tewari KS Landrum LM Pemetrexed and cisplatin for the treatment of advanced, persistent, or recurrent carcinoma of the cervix: a limited access phase II trial of the gynecologic oncology group. J Clin Oncol (2014) 32(25):2744–9. 10.1200/JCO.2013.54.7448 25071133 PMC4145184

[10] Hakansson P Hofer A Thelander L. Regulation of mammalian ribonucleotide reduction and dNTP pools after DNA damage and in resting cells. J Biol Chem (2006) 281(12):7834–41. 10.1074/jbc.M512894200 16436374

[11] Reichard P Eliasson R Ingemarson R Thelander L. Cross-talk between the allosteric effector-binding sites in mouse ribonucleotide reductase. J Biol Chem (2000) 275(42):33021–6. 10.1074/jbc.M005337200 10884394

[12] Radivoyevitch T Kunos CA. On model ensemble analyses of nonmonotonic data. Nucleosides Nucleotides Nucleic Acids (2012) 31(2):147–56. 10.1080/15257770.2011.644370 22303993 PMC3307047

[13] Kunos CA Chiu SM Pink J Kinsella TJ. Modulating radiation resistance by inhibiting ribonucleotide reductase in cancers with virally or mutationally silenced p53 protein. Radiat Res (2009) 172(6):666–76. 10.1667/RR1858.1 19929413 PMC2818576

[14] Kunos CA Radivoyevitch T Pink J Chiu SM Stefan T Jacobberger J Ribonucleotide reductase inhibition enhances chemoradiosensitivity of human cervical cancers. Radiat Res (2010) 174(5):574–81. 10.1667/RR2273.1 20954859 PMC3529744

[15] Kunos CA Ferris G Pyatka N Pink J Radivoyevitch T. Deoxynucleoside salvage facilitates DNA repair during ribonucleotide reductase blockade in human cervical cancers. Radiat Res (2011) 176(4):425–33. 10.1667/RR2556.1 21756082 PMC3191339

[16] Barker CA Burgan WE Carter DJ Cerna D Gius D Hollingshead MG In vitro and in vivo radiosensitization induced by the ribonucleotide reductase inhibitor triapine (3-aminopyridine-2-carboxaldehyde-thiosemicarbazone). Clin Cancer Res (2006) 12(9):2912–8. 10.1158/1078-0432.CCR-05-2860 16675588

[17] Finch RA Liu MC Cory AH Cory JG Sartorelli AC. Triapine (3-aminopyridine-2-carboxaldehyde thiosemicarbazone; 3-AP): an inhibitor of ribonucleotide reductase with antineoplastic activity. Adv Enzyme Regul (1999) 39:3–12. 10.1016/S0065-2571(98)00017-X 10470363

[18] Finch RA Liu M Grill SP Rose WC Loomis R Vasquez KM Triapine (3-aminopyridine-2-carboxaldehyde-thiosemicarbazone): a potent inhibitor of ribonucleotide reductase activity with broad spectrum antitumor activity. Biochem Pharmacol (2000) 59(8):983–91. 10.1016/S0006-2952(99)00419-0 10692563

[19] Chabes A Georgieva B Domkin V Zhao X Rothstein R Thelander L. Survival of DNA damage in yeast directly depends on increased dNTP levels allowed by relaxed feedback inhibition of ribonucleotide reductase. Cell (2003) 112(3):391–401. 10.1016/S0092-8674(03)00075-8 12581528

[20] Feun L Modiano M Lee K Mao J Marini A Savaraj N Phase I and pharmacokinetic study of 3-aminopyridine-2-carboxaldehyde thiosemicarbazone (3-AP) using a single intravenous dose schedule. Cancer Chemother Pharmacol (2002) 50(3):223–9. 10.1007/s00280-002-0480-0 12203104

[21] Murren J Modiano M Clairmont C Lambert P Savaraj N Doyle T Phase I and pharmacokinetic study of triapine, a potent ribonucleotide reductase inhibitor, administered daily for five days in patients with advanced solid tumors. Clin Cancer Res (2003) 9(11):4092–100. 14519631

[22] Wadler S Makower D Clairmont C Lambert P Fehn K Sznol M. Phase I and pharmacokinetic study of the ribonucleotide reductase inhibitor, 3-aminopyridine-2-carboxaldehyde thiosemicarbazone, administered by 96-hour intravenous continuous infusion. J Clin Oncol (2004) 22(9):1553–63. 10.1200/JCO.2004.07.158 15117978

[23] Kunos CA Waggoner S Von Gruenigen V Eldermire E Pink J Dowlati A Phase I trial of pelvic radiation, weekly cisplatin, and 3-aminopyridine-2-carboxaldehyde thiosemicarbazone (3-AP, NSC #663249) for locally advanced cervical cancer. Clin Cancer Res (2010) 16(4):1298–306. 10.1158/1078-0432.CCR-09-2469 20145183 PMC2822897

[24] Kunos CA Radivoyevitch T Waggoner S Debernardo R Zanotti K Resnick K Radiochemotherapy plus 3-aminopyridine-2-carboxaldehyde thiosemicarbazone (3-AP, NSC #663249) in advanced-stage cervical and vaginal cancers. Gynecol Oncol (2013) 130(1):75–80. 10.1016/j.ygyno.2013.04.019 23603372 PMC4260802

[25] Kunos CA Sherertz TM. Long-term disease control with triapine-based radiochemotherapy for patients with stage IB2-IIIB cervical cancer. Front Oncol (2014) 4:184. 10.3389/fonc.2014.00184 25105092 PMC4109518

[26] Therasse P Arbuck SG Eisenhauer EA Wanders J Kaplan RS Rubinstein L New guidelines to evaluate the response to treatment in solid tumors. European Organization for Research and Treatment of Cancer, National Cancer Institute of the United States, National Cancer Institute of Canada. J Natl Cancer Inst (2000) 92(3):205–16. 10.1093/jnci/92.3.205 10655437

[27] Basha MT Rodriguez C Richardson DR Martinez M Bernhardt PV. Kinetic studies on the oxidation of oxyhemoglobin by biologically active iron thiosemicarbazone complexes: relevance to iron-chelator-induced methemoglobinemia. J Biol Inorg Chem (2014) 19(3):349–57. 10.1007/s00775-013-1070-9 24317633

[28] Papadimitriou CA Sarris K Moulopoulos LA Fountzilas G Anagnostopoulos A Voulgaris Z Phase II trial of paclitaxel and cisplatin in metastatic and recurrent carcinoma of the uterine cervix. J Clin Oncol (1999) 17(3):761–6. 10.1200/JCO.1999.17.3.761 10071264

[29] Murren J Modiano M Plezia P Doyon A Bagulho T Johnson B A phase I study of 3-aminopyridine-2-carboxaldehyde thiosemicarbazone (triapine) in combination with cisplatin (CDDP). Proc Am Soc Clin Oncol (2003) 22:160a; abstract 643.

[30] Yen Y Margolin K Doroshow J Fishman M Johnson B Clairmont C A phase I trial of 3-aminopyridine-2-carboxaldehyde thiosemicarbazone in combination with gemcitabine for patients with advanced cancer. Cancer Chemother Pharmacol (2004) 54(4):331–42. 10.1007/s00280-004-0821-2 15148626

[31] Yee KW Cortes J Ferrajoli A Garcia-Manero G Verstovsek S Wierda W Triapine and cytarabine is an active combination in patients with acute leukemia or myelodysplastic syndrome. Leuk Res (2006) 30(7):813–22. 10.1016/j.leukres.2005.12.013 16478631

[32] Kunos C Radivoyevitch T Abdul-Karim FW Fanning J Abulafia O Bonebrake AJ Ribonucleotide reductase inhibition restores platinum-sensitivity in platinum-resistant ovarian cancer: a Gynecologic Oncology Group Study. J Transl Med (2012) 10:79. 10.1186/1479-5876-10-79 22541066 PMC3403898

[33] Chabes A Thelander L DNA building blocks at the foundation of better survival. Cell Cycle (2003) 2(3):171–3. 10.4161/cc.2.3.354 12734415

[34] Ratner ES Zhu YL Penketh PG Berenblum J Whicker ME Huang PH Triapine potentiates platinum-based combination therapy by disruption of homologous recombination repair. Br J Cancer (2016) 114(7):777–86. 10.1038/bjc.2016.54 26964031 PMC4984868

